# Dyspnoe und Ödeme bei einem 79-jährigen Patienten

**DOI:** 10.1007/s00108-022-01358-0

**Published:** 2022-06-23

**Authors:** Frederic Schwarz, Karin Klingel, Simon Greulich, Meinrad Gawaz

**Affiliations:** 1 Medizinische Klinik III (Kardiologie und Angiologie), Universitätsklinikum Tübingen, Otfried-Müller-Str. 10, 72076 Tübingen, Deutschland; 2 Kardiopathologie, Institut für Pathologie und Neuropathologie, Universitätsklinikum Tübingen, Tübingen, Deutschland

**Keywords:** ATTR-Amyloidose, Kardiale Beteiligung, Restriktive Kardiomyopathie, Knochenszintigraphie, Biopsie, TTR-Amyloidosis, Cardiac involvement, Restrictive cardiomyopathy, Bone scintigraphy, Biopsy

## Abstract

**Hintergrund:**

Die Transthyretin-Amyloidose (ATTR-Amyloidose) führt zur Ablagerung von unlöslichen Fibrillen im Interstitium der betroffenen Organe. Eine kardiale Beteiligung kann sich durch Dyspnoe, Ödeme, Rhythmusstörungen bis hin zur manifesten Herzinsuffizienz und Tod äußern.

**Fallbericht:**

Ein 79-jähriger Mann stellte sich mit Dyspnoe sowie Gewichtszunahme vor. In der Echokardiographie Hypertrophie bei restriktiver Kardiomyopathie. In der Knochenszintigraphie Tracer-Mehranreicherung, hochverdächtig auf eine ATTR-Amyloidose, welche mittels Biopsie bestätigt werden konnte.

**Schlussfolgerungen:**

Die Diagnose einer kardialen ATTR-Amyloidose stellt für den Kliniker eine Herausforderung dar und setzt dessen erhöhte Aufmerksamkeit voraus. Die Diagnosestellung sollte strukturiert erfolgen unter Einbeziehung von Labor, bildgebenden Verfahren sowie Myokardbiopsie.

## Abstract (en)

**Background:**

Transthyretin(TTR)-amyloidosis (hereditary or wild-type) is characterized by deposition of misfold, insoluble amyloid fibrils in the interstitial space, leading to dysfunction of the involved organs. Cardiac involvement may vary, ranging from dyspnea, edema, and arrhythmia to overt heart failure and death.

**Case report:**

A 79-year-old Caucasian male presented with dyspnea, edema, and weight gain. Echocardiography revealed left ventricular wall thickening and restrictive cardiomyopathy. Bone scintigraphy revealed abnormal cardiac tracer uptake consistent with cardiac TTR-amyloidosis, which could be confirmed by endomyocardial biopsy.

**Conclusion:**

The diagnosis of TTR-amyloidosis is challenging for the clinician and requires their heightened awareness. Definitive diagnosis needs a structured approach including laboratory and imaging findings combined with endomyocardial biopsy.

## Anamnese

Ein 79-jähriger Patient wird wegen progredienter Dyspnoe (NYHA-Grad III–IV) und intermittierenden thorakalen Beschwerden aufgenommen. Zudem berichtet der Patient über eine massive Gewichtszunahme (8 kg in den letzten 4 Tagen) sowie Beinödeme. Vorbekannt sind eine diffuse koronare 3‑Gefäß-Erkrankung mit Z. n. Intervention im Bereich der RCA vor 1,5 Jahren (DES-Implantation) sowie ein Vorhofflimmern und Z. n. DDD-Schrittmacher-Implantation bei intermittierendem AV-Block III°. An kardiovaskulären Risikofaktoren bestehen eine arterielle Hypertonie und Hypercholesterinämie.

## Klinischer Befund

Reduzierter Allgemeinzustand bei adipösem Ernährungszustand. Der Blutdruck betrug 120/70 mm Hg, die Herzfrequenz 60/min. Die Körpertemperatur wurde mit 36,0 °C gemessen, es bestand eine erhöhte Atemfrequenz von 20/min. Die Herztöne imponierten auskultatorisch rhythmisch, 3/6-Systolikum mit p.m. über dem 5. Interkostalraum medioklavikulär. Pulmonal vesikuläres Atemgeräusch, rechts basal abgeschwächt. Die Jugularvenen waren deutlich gestaut. Das Abdomen war prall mit regelrechten Darmgeräuschen in allen vier Quadranten. Beidseits ausgeprägte Beinödeme.

## Laborbefund

Erhöhung des NT-proBNP von 6321 ng/l (Normbereich < 300 ng/l) und Troponin I mit 0,14 µg/l (Normwert < 0,04 µg/l). Bei normwertigen Transaminasen imponierten eine Erhöhung der y‑Glutamyltransferase von 476 U/l (Normwert 10–66 U/l) und ein Anstieg der alkalischen Phosphatase von 216 U/l (Normwert 40–130 U/l). Die Infektionsparameter zeigten eine leichte Lymphopenie von 18,7 % (Normwert 20–45 %), keine erhöhte Leukozytenzahl 8970/µl (Normwert 3800–10.300 pro µl) und einen leichten Anstieg des C‑reaktiven Proteins von 0,61 mg/dl (Normwert max. 0,5 mg/dl). Hb lag bei 10,2 g/dl (Normwert 14–18 g/dl), Kreatinin bei 1,1 mg/dl (Normwert 0,6–1,1 mg/dl), die GFR betrug 64,6 ml/min/1,73 m^2^ (Referenz: min. > 60 ml/min/1,73 m^2^), Calcium 2,2 mmol/l (Normwert 2,1–2,6 mmol/l).

Die weiterführende Labordiagnostik zeigte vermehrte freie Leichtketten vom Typ kappa von 33,30 mg/dl (Normbereich 6,7–22,4 mg/dl) und lambda von 35,60 mg/dl (Normbereich 8,3–27,00 mg/dl), der Quotient lag mit 0,94 im Referenzbereich (0,31–1,56). Eine ergänzende Serum- bzw. Immunfixation zeigte unauffällige Ergebnisse.

## EKG

**Figure 1:**
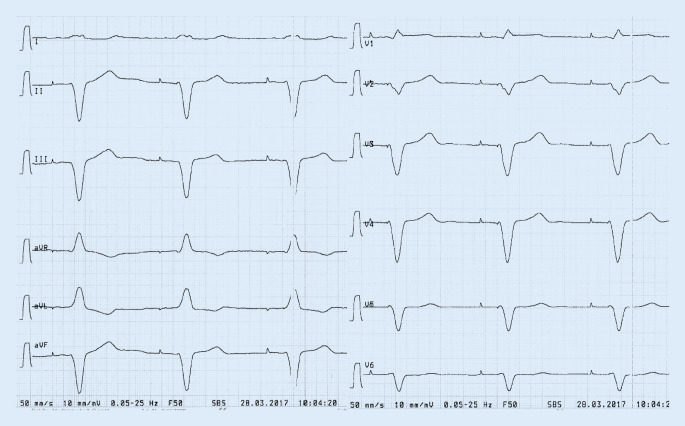

Schrittmacherstimulation, Frequenz 60/min, s. Abb. 1.

## Röntgenaufnahme des Thorax in zwei Ebenen

**Figure 2:**
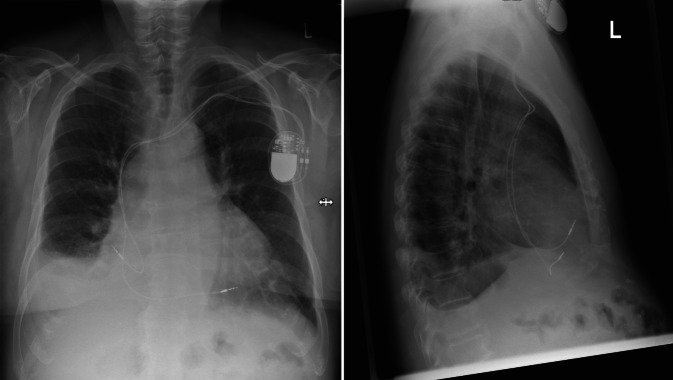

Diese zeigte ein verbreitertes, mittelständiges Mediastinum. Kein Nachweis umschriebener Infiltrate. Keine höhergradige pulmonalvenöse Volumenbelastung. Auslaufender Pleuraerguss rechts mit angrenzender Belüftungsstörung. Unverändert einliegender Schrittmacher mit Projektion der Sondenspitzen auf den rechten Vorhof und den Boden des rechten Ventrikels, Abb. 2.

## Transthorakale Echokardiographie

**Figure 3:**
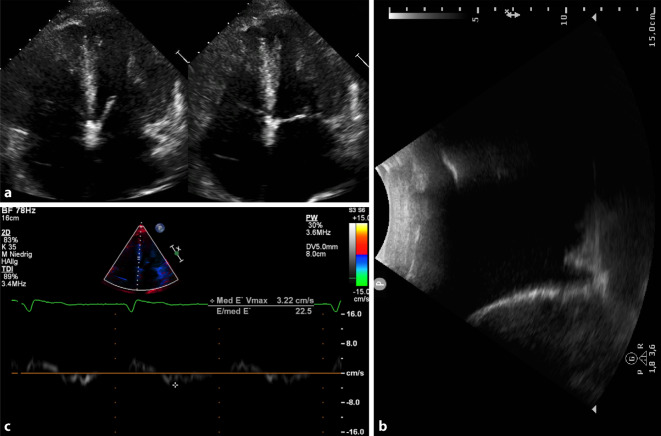

Die Echokardiographie zeigte einen konzentrisch hypertrophierten linken Ventrikel mit mittelgradig diffus eingeschränkter Pumpfunktion, deutlich dilatierte Vorhöfe und eine auffällige Myokardtextur vereinbar mit kardialer Speichererkrankung, Abb. 3. Es bestand eine ausgeprägte diastolische Dysfunktion (E/eʼ = 23) mit restriktivem Füllungsmuster im PW-Doppler. Aortenklappeninsuffizienz Grad I–II, Mitralklappeninsuffizienz Grad I–II und Trikuspidalklappeninsuffizienz Grad II mit einem PAP_sys_ von 23 mm Hg + ZVD. Nebenbefundlich wurde ein massiver Pleuraerguss rechts festgestellt, kein Perikarderguss.

## Verdachtsdiagnose (aufgrund der klinischen, laborchemischen Untersuchung und der Bildgebung)

Kardiale Amyloidose

## Knochenszintigraphie

**Figure 4:**
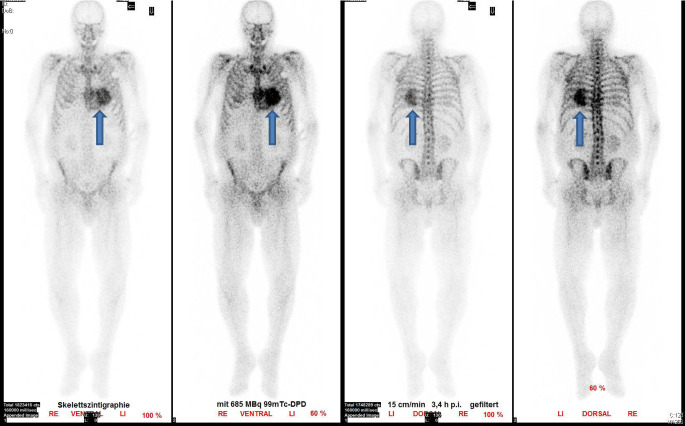

Zur Erhärtung der Verdachtsdiagnose kardiale Amyloidose wurde eine Knochenszintigraphie durchgeführt, welche eine massive Traceranreicherung (Perugini-Score 3) im nahezu gesamten linksventrikulären Myokard, passend zu einer Transthyretinamyloidose, zeigte; Abb. 4.

## Herzkatheter und Myokardbiopsie

**Figure 5:**
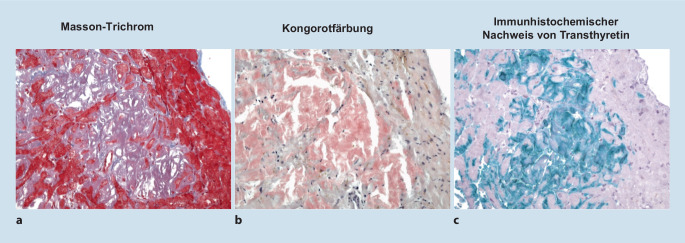

Die Koronarien zeigten keine interventionspflichtigen Stenosen. In den Endomyokardbiopsien fand sich in den Kongorotfärbungen eine ausprägte Anfärbung der überwiegend fokal im Interstitium eingelagerten Amyloidfibrillen. Die nachfolgend durchgeführten immunhistologischen Färbungen zeigten eine ausgeprägte positive Reaktion für Transthyretin als Beleg für eine ATTR-Amyloidose, Abb. 5.

## Diagnose

- In der Gesamtschau der Befunde (Klinik, Bildgebung und histologische Untersuchung) stellten wir die Diagnose einer kardialen ATTR-Amyloidose.

## Therapie und Verlauf

Wegen der rasch progredienten Herzinsuffizienzsymptomatik wurde entsprechend eine Rekompensationstherapie und Intensivierung der diuretischen Therapie begonnen. Hierunter besserte sich der Zustand deutlich. Auch der Pleuraerguss war unter forcierter diuretischer Therapie deutlich rückläufig. Neben diuretischer Medikation wurde die Herzinsuffizienztherapie optimiert (ACE-Hemmer, Betablocker, Eplerenon). Zusätzlich trank der Patient täglich grünen Tee. Eine dezidierte neurologische Vorstellung zur Abklärung einer möglichen Polyneuropathie wurde in die Wege geleitet. Eine genetische Untersuchung zum Ausschluss einer erblichen ATTR-Amyloidose wurde von dem 79-jährigen Patienten nicht gewünscht.

## Diskussion

Unter Amyloidosen versteht man Proteinfehlfaltungskrankheiten, bei denen sich verschiedene vom Körper produzierte Eiweiße infolge einer Konformationsänderung als unlösliche fibrilläre Aggregate im Myokard ablagern. Dies geschieht bei den systemischen Formen meist extrazellulär, betroffen können prinzipiell alle Gewebe sein. Es werden verschiedene Formen unterschieden, am häufigsten ist die Leichtketten(AL)-Amyloidose, gefolgt von der Transthyretin(ATTR)-Amyloidose. Die Amyloid-A(AA)-Amyloidose tritt weitaus seltener auf. Weiterhin gibt es seltene Amyloidoseformen mit nichtkardialen Organmanifestationen und auch lokale Amyloidosen. Bei 90 % der Patienten mit AL-Amyloidose liegt als Ursache eine Plasmazelldyskrasie mit Expression einer monoklonalen Gammopathie (MG) vor; nur ca. 10 % sind an einem symptomatischen multiplen Myelom oder einem B‑Zell-Lymphom erkrankt [1].

## Erbliche ATTR-Amyloidose

Es gibt verschiedene Typen der erblichen Amyloidose, der bekannteste ist die ATTR-Amyloidose. Hierbei führen die Transthyretinablagerungen oftmals zunächst zu einer schweren peripheren und autonomen Neuropathie. Die klassischen Zeichen und Symptome sind Hyperästhesie, Impotenz, Obstipation, Durchfall, Gewichtsverlust, Inkontinenz, orthostatische Hypotonie etc. [2]. Bei myokardialer Ablagerung kommt es zur Entwicklung einer Kardiomyopathie, welche über Herzinsuffizienz und Herzrhythmusstörungen bis hin zum Tode führen kann. Mehr als 100 verschiedene Mutationen im Transthyretinprotein sind bekannt.

## Wildtyp-ATTR-Amyloidose/senile systemische Amyloidose

Diese Form der ATTR-Amyloidose tritt durch altersabhängige Prozesse (> 65 Jahre) auf. Die hierbei zugrunde liegenden Pathomechanismen sind bislang unbekannt. Überwiegend sind Männer betroffen und in > 90 % liegt eine kardiale Beteiligung vor. Eine gehäufte Assoziation findet sich zu Karpaltunnelsyndrom, Spinalkanalstenose oder atraumatischer Bizepssehnenruptur. Die mittlere Lebenserwartung liegt bei 3,5 Jahren. Ca. 50 % der Patienten haben Vorhofflimmern, darüber hinaus ist 1/3 der Patienten Schrittmacherträger.

## Endomyokardbiopsie und Knochenszintigraphie

Die Endomyokardbiopsie stellt den Goldstandard für die Diagnose einer kardialen Amyloidose dar, da sie sicher zwischen ATTR-, AL- und AA-Amyloidosen differenzieren kann und zudem eine Charakterisierung weiterer pathologischer Befunde (Inflammation, Fibrose) ermöglicht. Sie hat jedoch den Nachteil der Invasivität. Bei V. a. kardiale Amyloidose und Abwesenheit einer monoklonalen Gammopathie (keine pathologische Freie-Leichtketten-Ratio, negative Immunfixation im Serum und Urin) hat die Knochenszintigraphie mit ^99m^Tc-Phosphat für die Diagnose einer ATTR-Amyloidose eine Sensitivität von > 99 % und Spezifität von 86 %. Hierbei gilt, je stärker die Traceranreicherung im Myokard ausgeprägt ist (Perugini-Score 2 und 3), desto höher die Wahrscheinlichkeit für eine ATTR-Amyloidose [3–5].

## Kardiales MRT – gute Gewebecharakterisierung

Außer der Knochenszintigraphie kann auch die kardiale MRT (CMR) aufgrund ihrer exzellenten Gewebecharakterisierung Veränderungen des Herzmuskels, wie sie bei der Amyloidose im Rahmen der Proteinablagerung auftreten können, gut abbilden. Neben der etablierten Late-gadolinium-enhancement(LGE)-Technik, welche in erster Linie fokale fibrotische Prozesse mit hoher Genauigkeit abbilden kann, lassen sich mittels aktueller CMR-Techniken der Gewebecharakterisierung (T1/T2-Mapping und vor allem Bestimmung des Extrazellulärvolumens [ECV], welches typischerweise bei Amyloidose deutlich vergrößert ist) zusätzlich diffuse myokardiale Veränderungen quantifizieren [6, 7]. Vorteilhaft gegenüber der Knochenszintigraphie ist die fehlende Strahlenbelastung des Patienten, nachteilig ist die fehlende Unterscheidbarkeit zwischen den einzelnen Amyloidoseformen.

## Therapie der ATTR-Amyloidose

Neben der Optimierung der Herzinsuffizienztherapie (cave: bis auf Diuretika werden die gängigen Herzinsuffizienzmedikamente teilweise sehr schlecht hämodynamisch toleriert) stehen neue zugelassene Therapieoptionen bei kardialer Amyloidose zur Verfügung. Seit Februar 2020 ist Tafamidis, ein oral angewendeter Inhibitor der TTR-Tetramer-Dissoziation zur Behandlung der kardialen Wildtyp-ATTR- bzw. hereditären ATTR-Amyloidose zugelassen. Tafamidis bindet an der Thyroxinbindestelle des TTR-Tetramers, stabilisiert dieses und verhindert die Fibrillenbildung. Die Behandlung mit Tafamidis wird laut aktueller „European Society of Cardiology guideline“ empfohlen für Patienten mit genetisch bestätigter hereditärer ATTR- oder Wildtyp-ATTR-Kardiomyopathie und NYHA-Stadium I oder II zur Reduktion von Symptomen, kardiovaskulär bedingten Hospitalisierungen und Gesamtmortalität [8]. Weitere Therapeutika, welche bereits für die hereditäre ATTR mit Polyneuropathie zugelassen sind, sind aktuell noch nicht zugelassen. Bei hereditärer kardialer ATTR-Amyloidose ist eine Lebertransplantation ein kurativer Ansatz, jedoch kommt bei bestehender Herzinsuffizienz meist nur eine kombinierte Leber-Herz-Transplantation bzw. eine isolierte Herztransplantation infrage, zudem gibt es Berichte über Wildtyp-ATTR Ablagerungen auch bei Patienten mit „eigentlich“ hereditärer ATTR-Amyloidose.

## Fazit für die Praxis

Die Diagnose einer kardialen Amyloidose erfordert ein standardisiertes und interdisziplinäres Vorgehen. Klinik, EKG und Echokardiographie können bereits hinweisend sein, müssen aber auch durch Labor sowie Knochenszintigraphie/CMR bis hin zur Myokardbiopsie ergänzt werden. Neuere Therapieansätze sollten zu einer exakten Diagnose der ATTR-Amyloidose motivieren.
